# Association of Oral Health Literacy and Dental Visitation in an Inner-City Emergency Department Population

**DOI:** 10.3390/ijerph15081748

**Published:** 2018-08-15

**Authors:** Emmett Henderson, Preeti Dalawari, Jennifer Fitzgerald, Leslie Hinyard

**Affiliations:** 1 Center for Health Outcomes Research, Saint Louis University, Saint Louis, MO 63104, USA; Emmett.henderson@health.slu.edu (E.H.); leslie.hinyard@health.slu.edu (L.H.); 2 Department of Surgery, Division of Emergency Medicine, Saint Louis University, Saint Louis, MO 63110, USA; Preeti.dalawari@health.slu.edu

**Keywords:** oral health literacy, emergency medicine, oral health, public health

## Abstract

To examine the association between oral health literacy (OHL) with sociodemographic variables and dental visitation in adults presenting to an urban emergency department (ED). *Methods:* This was a cross-sectional study of a convenience sample of 556 adults aged 18–90. Interview data from the study were used to collect self-reported sociodemographic characteristics and dental visitation history. The OHL of the study participants was measured using the Health Literacy in Dentistry scale (HeLD-14), and the score was dichotomized into low and high OHL. Bivariate associations between sociodemographic variables and OHL were conducted using chi-square tests, and logistic regression was used to examine the association between OHL and dental visitation within the past year. *Results:* Sixty percent of participants reported having visited a dentist within the past year. Over two-thirds of the sample was classified as having low OHL. Low OHL was more common in non-White races, less-educated, single, unemployed, and lower-income individuals, and those without a primary care physician or dental insurance (*p* < 0.05). Patients with low oral health literacy were 39% less likely to have visited the dentist in the past year (OR = 0.61; 95% CI 0.38, 0.96). *Conclusions:* This study highlights significant disparities in OHL. Interventions targeted toward the unique needs of underserved populations should be developed to improve health outcomes.

## 1. Introduction

In the past decade, there has been a growing body of literature dedicated to resolving low health literacy in the United States. Health literacy was defined by the World Health Organization (WHO) as “the cognitive and social skills which determine the motivation and ability of individuals to gain access to, understand, and use the information in ways which promote and maintain good health [1]”. Since health literacy is such an important determinant of health [2], it is concerning that only an estimated 12% of adults in the United States have proficient health literacy [3]. Individuals with low health literacy have increased adverse health outcomes, such as diabetes and emergency department (ED) utilization, as well as a decreased use of potentially life-saving services such as preventive screenings [4]. Because of these adverse health outcomes, estimated costs resulting from low health literacy range between $106 billion and $238 billion annually [5].

Although the evidence regarding the adverse effects of low health literacy is abundant, there is a paucity of research linking low oral health literacy (OHL) to negative health outcomes. The available research associates low OHL with poor outcomes including oral health status [6,7], dental neglect [8], sporadic dental attendance [9], and likelihood to fail appointments [10]. In the efforts to understand OHL, many ethnic [11,12,13,14,15] and sociodemographic [16,17,18,19] inequalities have emerged. Like health literacy, estimates of OHL in the United States population are low. In a 2007 study, 29% of respondents had a low OHL [7].

Despite the increasing knowledge base, conclusions about the association between OHL and regular dental utilization are conflicting. Previous studies have shown OHL to be weakly correlated with oral health-related quality of life [20] and highly correlated with recent dental visits [21]. However, two studies have also found no association between OHL and dental utilization [22,23]. Annual visits to dentists have steadily decreased from 63.6% in 2007 to 61.8% in 2010 in adults aged 21–64. The lack of insurance and poverty have been recognized as contributing factors [24].

Another factor associated with decreased dental utilization is the increasing use of the ED for non-traumatic dental problems [25]. There are about 2 million annual ED visits in the United States for non-traumatic dental complaints, accounting for about 1.5–2.5% of all ED visits [26]. These ED visits are costly and consist primarily of palliative care [27]. Socioeconomically disadvantaged individuals are at increased risk for repeated ED dental visits because of barriers to accessing oral health care [27]. Efforts to reduce such barriers include insurance benefit expansion and care coordination [28]. However, increasing OHL may provide another avenue to increase the rate of dental care utilization outside of the ED.

Given the conflicting results, there is a need to build upon previous work regarding the association between OHL and dental utilization. The purposes of this study are (1) to determine the association between OHL and dental visitation in adults aged 18 or older presenting in an urban ED; and (2) to identify the association between sociodemographic variables and OHL.

## 2. Methods

### 2.1. Study Population and Recruitment

This manuscript is the result of a cross-sectional analysis of the baseline data from a prospective institutional review board-approved study designed to examine general health and oral health literacy, oral health care practices, oral health knowledge, and access to primary care doctors and dental providers. Participants made up a convenience sample of adults presenting to an inner-city ED. The site was an urban academic tertiary care center with an annual volume of 48,000 ED visits and an admission rate of 30%. The center was in an urban area with a racial/ethnic mix of African Americans (50%), non-Hispanic Whites (46%), and “Other” (4%). Data was collected from January to December 2016 when research associates were available. Research associates were instructed to approach as many patients and their visitors as possible to check eligibility for the study. Inclusion criteria included (a) age 18–90; (b) no acute distress; and (c) able to speak and understand English. Exclusion criteria included (a) sexual assault victims; (b) incarcerated persons/prisoners or patients under arrest; (c) psychiatric patients or homicidal/suicidal patients; (d) patients incapacitated because of drugs or alcohol; (e) patients unable to communicate effectively (e.g., hearing-impaired, intoxicated, or violent patients); and (f) patients in distress/extremis (e.g., those in resuscitation areas). Verbal consent was obtained from study participants, and responses were recorded anonymously. Oral health kits (comprised of a toothbrush, fluoride toothpaste, floss, and American Dental Association recommendations for proper oral health care) were given after survey completion. Study participants received follow-up phone calls two weeks after their visit to the ED. This study is an analysis of the baseline data obtained.

### 2.2. Measures

Demographic characteristics included sex, age, race/ethnicity, education, marital status, employment status, and household income. Age was measured in years and categorized as 18–40, 41–65, and ≥65. Race/ethnicity was classified as White, Black, or “Other” based on self-reported race/ethnicity. Education was classified as less than high school, high school graduate, and college graduate. Marital status was dichotomized into single or married/domestic partner. Employment status was categorized as full-time/part-time, retired, or unemployed/disabled. Household income was classified as <$19,999, $20,000–49,999, $50,000–74,999, or ≥$75,000. The presence of dental insurance was dichotomized into yes or no.

A current dental visit was classified as having seen a dentist in the past 12 months. Oral health literacy (OHL) was measured using the Health Literacy in Dentistry scale (HeLD-14) [29]. HeLD-14 is a short-form, 14-item version of the 29-item Health Literacy in Dentistry (HeLD-29) instrument. The questions were designed to assess oral health literacy in seven domains: communication, understanding, receptivity, utilization, support, financial resources, and access. Participants were asked their ability to perform tasks such as “pay attention to your dental and oral health needs, fill out dental forms or carry out instructions from a dentist”. Each item was ranked on a 5-point Likert scale from 1 (without any difficulty) to 5 (unable to do so). The HeLD-14 scale scores ranged from 0 to 56, where higher scores indicated low oral health literacy [29]. Because of a bimodal distribution in the HeLD-14 scores, OHL was dichotomized into low (HeLD-14 score >19) and high (HeLD-14 score ≤19) for analysis [30].

### 2.3. Statistical Analyses

Descriptive statistics for sociodemographic characteristics and dental use were calculated. Bivariate comparisons were made using chi-square and logistic regression to assess the association between OHL and dental visitation within the last 12 months, adjusting for other sociodemographic factors. All analyses were conducted using the SAS System version 9.4 (SAS Institute Inc., Cary, NC, USA). All associations were deemed statistically significant with *p* < 0.05.

## 3. Results

Table 1 presents the demographics of the study sample. Among the 556 respondents, 56% were female, and half were aged 41–65 (50.0%). There were, proportionally, slightly more Black than White study participants (47% and 46%, respectively). The majority indicated that high school graduate was their highest level of education (61%) and were employed either full- or part-time (45%). Nearly 64% of participants indicated that their yearly income was less than $49,999. Slightly over half of respondents had dental insurance (54%), and 61% reported visiting the dentist within the past year.

Table 2 shows the bivariate associations between OHL and time since last dental visit. Patients with high OHL were more likely to have seen a dentist in the past 12 months than those with low OHL (*p* < 0.001). OHL was also associated with race/ethnicity (*p* = 0.03), education (*p* < 0.001), marital status (*p* = 0.01), employment status (0.003), income (<0.001), having a primary care physician (*p* = 0.003), and dental insurance (*p* < 0.001).

Multiple logistic regression was used to examine the association between OHL and time since last dental visit, adjusting for sociodemographic factors (Table 3). ED patients with low OHL were 39% less likely to be current with dental visits compared to those with high OHL (OR = 0.61, 95% CI 0.38, 0.96) after adjusting for sex, age, race, education, marital status, employment status, income, having a primary care physician, and dental insurance. Compared to college graduates, patients with less than high school education and high school graduates were less likely to be current with dental visits (OR = 0.33, 95% CI 0.17, 0.63 and OR = 0.16, 95% CI 0.07, 0.37, respectively). Income was also associated with being current with dental visits, with those earning less than $75,000 less likely to be current. Patients with a primary care physician were 3.33 times more likely to be current with dental visits compared to those without a main source of primary care (OR = 3.33, 95% CI 1.78, 6.21), and those with dental insurance were also more likely to be current with dental care (OR = 3.75, 95% CI 2.45, 5.74). There was no statistically significant association between sex, age, race, marital status, or employment status and a recent dental visit.

## 4. Discussion

This study is among the first to examine the association between OHL and dental visitation in a diverse sample of patients from an emergency department. Within the sample, we observed several sociodemographic disparities in OHL. In an unadjusted analysis, lower OHL was found in racial minorities, those with lower education, unemployed or disabled individuals, single individuals, and those without a regular source of primary care or without dental insurance. These results are not surprising given that disparities among racial and ethnic minorities, men, low-income, and uninsured individuals are well documented [11,12,13,14,15,16,17,18,19]. Prior studies have reported on the effectiveness of educational interventions for improving oral health [31]. It is possible that most existing interventions have not sufficiently addressed the unique needs of health disparate populations. A pilot intervention targeted toward African American men was successful in increasing oral health literacy in this underrepresented group [15]. Thus, developing more culturally sensitive educational interventions may help to reduce current disparities in oral health literacy.

Consistent with a prior analysis, 54.1% of participants in this study reported visiting a dentist within the past year. Wall et al. found that 61.8% of non-elderly adults in 2010 used dental services at least once a year—a decrease from 66.8% in 2000. In contrast, Wall et al. reported in the same study that in 2010, 69.6% of elderly adults used dental services at least once a year [24]. Future research would benefit from similar stratification by age group. A similar study by Kenney et al. found that in 2010, 60.8% of non-elderly adults had visited a dentist within the past year [32]. Nearly a decade after the publication of these results, our study suggests that dental utilization has not increased. The lack of dental coverage under Medicaid [33] and the Affordable Care Act (ACA) [34] as well as a decrease in dental benefits provided by employers [35] may be contributing to the inability to improve dental utilization rates.

In addition to differences among sociodemographic groups, we also found lower levels of OHL in those who had not visited a dentist within the past year. The presence or absence of an association between OHL and dental utilization appears to be population-specific. Jamieson et al. found that OHL was lower for American Indians who had not visited the dentist in the past year. However, there was no such association for indigenous Australians [36]. In a random sample of adults in Iran, Naghibi Sistani et al. found an association between higher levels of OHL and dental visits within the past six months [21]. Furthermore, two studies using a multi-racial, community-based sample of women from North Carolina found no association between OHL and dental care visits [8,22]. Future research in the United States should aim to use a nationally representative, population-based study to determine whether the association between OHL and dental visitation differs between population sub-groups. 

To date, most studies that evaluated the association between OHL and dental visits sampled participants from a non-clinical setting. In the United States, the studies that sampled from an outpatient clinic found no association between OHL and dental visits [8,22]. Similarly, another study conducted in a non-clinical setting in the United States found no association [23]. The association found in this study may be explained by the differences in the assessment location. Because this study involved a sample of subjects in an ED setting, these individuals may also be more likely to use similar resources for dental-related complaints.

The association between OHL and dental visitation may also be instrument-specific. Jones et al. and Burgette et al. found no association using the REALD-30 [22,29]. In this study, we found an association using the HeLD-14. While the REALD-30 has been widely used and tested [6], the HeLD-14 was developed in part because of a reported dissatisfaction when using the REALD-30 [29]. Furthermore, REALD-30 is a word recognition scale, whereas HeLD-14 is a Likert-type scale. It is possible that the two scales are measuring different aspects of oral health literacy. Since HeLD-14 has only been used in small samples, continued efforts should be made to measure OHL using HeLD-14 in a larger sample of subjects.

## 5. Limitations

This study has several limitations. First, the data were collected from a non-probability convenience sample of adults in an inner-city ED. Although our sample was composed of a diverse group of patients, a generalizability beyond urban, English-speaking, and non-institutionalized adults is not possible. Future research should utilize population-based probability samples to evaluate OHL. Furthermore, OHL scales should be validated in non-English-speaking populations to elucidate further disparities in underrepresented populations. Second, this study was cross-sectional by design, and because of the lack of temporality, a causal inference cannot be estimated. Surprisingly, a high proportion of patients had dental insurance and a primary care physician in this sample. It is possible that there is a selection bias in this study, especially since research associates were not available during overnight hours. Finally, social desirability in response to the demographic and dental visitation questions may have led to an overestimation of the number of individuals who visited the dentist in the past year. Caution should be taken when interpreting the results of self-reported health behavior.

Despite such limitations, our study is one of the few in the literature to examine the association of oral health literacy and dental visitation. Interventions targeted toward the unique needs of underserved populations should be developed. Given that oral health literacy and primary prevention for oral health require access to oral health services, reducing the logistic and economic barriers to oral health services is a strong strategy for improving both OHL and dental visitation. Though the ED is likely not the ideal setting for educational interventions, it does provide an optimal setting for identifying underserved patients with additional oral health needs. It is possible that the ED can serve as the point of contact for identifying patients in need of oral health education or services for referral to additional resources, including community-based dental clinics. It has been suggested that providing basic oral health education and screening in primary care locations, particularly Federally Qualified Health Centers (FQHCs), with a referral to other low-cost dental services may improve oral health outcomes for low-income patients [37,38]. In fact, the Institute of Medicine 2011 report *Oral Health in America* recommends promoting oral health education through all state and local health systems, and not just dental providers [39]. Alternatively, the use of community-based lay health advisors has demonstrated improvements in OHL in Taiwanese children—a strategy that holds the potential for effective improvements in OHL in other populations [40]. Regardless of the mechanism, pre-test and post-test designs should be implemented to evaluate the effectiveness of such interventions.

## 6. Conclusions

In this emergency department sample, participants with low oral health literacy scores were less likely to keep current with dental visits, after controlling for sociodemographic factors. This study may influence future practice by identifying a strategy to increase dental visits through increased OHL. Additionally, this study may highlight underserved populations which may benefit from more targeted interventions.

## Figures and Tables

**Table 1:** Demographic characteristics of the study sample, *N* = 556.

Demographic Characteristics	N (%)
Sex	
Female	313 (56.3)
Male	243 (43.7)
Age	
18–40	164 (29.5)
41–65	278 (50.0)
≥65	114 (20.5)
Race/Ethnicity	
White	254 (45.7)
Black	261 (46.9)
Other	41 (7.4)
Education	
Less than high school	92 (16.6)
High school graduate	342 (61.5)
College graduate	122 (21.9)
Marital status	
Single	361 (64.9)
Married or domestic partner	195 (35.1)
Employment status	
Full-time or part-time	251 (45.1)
Retired	127 (22.8)
Unemployed or disabled	178 (32.0)
Income	
<$19,999	227 (40.8)
$20,000–49,999	128 (23.0)
$50,000–74,999	48 (8.6)
≥$75,000	52 (9.4)
Missing	101 (18.2)
Primary care physician	
Yes	476 (85.6)
No	80 (14.4)
Dental insurance	
Yes	301 (54.1)
No	255 (45.9)
Time since last dental visit	
≤1 year	340 (61.2)
>1 year	216 (38.8)
Oral health literacy	
High	176 (31.6)
Low	380 (68.4)

**Table 2:** Bivariate comparison between oral health literacy (OHL) and sociodemographic variables.

Demographic Characteristics	Oral Health Literacy		*p* Value
	High	Low	*p* *
Sex			
Female	107 (60.8)	206 (54.2)	0.14
Male	69 (39.2)	174 (45.8)	
Age			
18–40	44 (25.0)	120 (31.6)	0.27
41–65	92 (52.3)	186 (49.0)	
≥65	40 (22.7)	74 (19.5)	
Race/ethnicity			
White	90 (51.1)	164 (43.2)	0.03
Black	80 (45.5)	181 (47.6)	
Other	6 (3.4)	35 (9.2)	
Education			
Less than high school	15 (8.5)	77 (20.3)	<0.001
High school graduate	180 (61.4)	234 (61.6)	
College graduate	53 (30.1)	69 (18.2)	
Marital status			
Single	101 (57.4)	260 (68.4)	0.01
Married or domestic partner	75 (42.6)	120 (31.6)	
Employment status			
Full-time or part-time	90 (51.1)	161 (42.4)	0.003
Retired	47 (26.7)	80 (21.0)	
Unemployed or disabled	39 (22.2)	139 (37.6)	
Income			
<$19,999	57 (32.4)	170 (44.7)	<0.001
$20,000–49,999	45 (25.6)	83 (21.8)	
$50,000–74,999	25 (14.2)	23 (6.01)	
≥$75,000	25 (14.2)	27 (7.1)	
Missing	24 (13.6)	77 (20.3)	
Primary care physician			
Yes	162 (92.1)	314 (82.6)	0.003
No	14 (7.9)	66 (17.4)	
Dental insurance			
Yes	117 (66.5)	184 (48.4)	<0.001
No	59 (33.5)	196 (51.6)	
Time since last dental visit			
≤1 year	132 (75.0)	208 (54.7)	<0.001
>1 year	44 (25.0)	172 (45.3)	

* Chi-square analysis.

**Table 3:** Logistic regression predicting a dental visit within the last 12 months.

Demographic Characteristics	Odds Ratio (95% CI)
Oral health literacy (ref = high)	0.61 (0.38, 0.96)
Sex (ref = male)	1.01 (0.67, 1.52)
Age	
18–40	1.47 (0.62, 3.50)
41–65	0.91 (0.44, 1.88)
≥65	Ref
Race/ethnicity	
White	Ref
Black	1.28 (0.82, 2.00)
Other	1.33 (0.60, 2.91)
Education	
Less than high school	0.33 (0.17, 0.63)
High school graduate	0.16 (0.07, 0.37)
College graduate	Ref
Marital status (ref = single)	0.90 (0.56, 1.44)
Employment status	
Full-time or part-time	Ref
Retired	1.03 (0.48, 2.19)
Unemployed or disabled	0.92 (0.54, 1.55)
Income	
<$19,999	0.15 (0.03, 0.74)
$20,000–49,999	0.14 (0.03, 0.67)
$50,000–74,999	0.13 (0.03, 0.67)
≥$75,000	Ref
Missing	0.10 (0.02, 0.51)
Primary care physician (ref = no)	3.33 (1.78, 6.21)
Dental insurance (ref = no)	3.75 (2.45, 5.74)
